# Striving for the Ideal: Narrative Positioning Analysis of Work Ability Support Experiences of Employees with Disabilities in Non-standard Employment

**DOI:** 10.1177/00469580251376226

**Published:** 2025-09-22

**Authors:** Hanna Keränen, Anu Järvensivu, Ritva Horppu

**Affiliations:** 1 Finnish Institute of Occupational Health, Työterveyslaitos, Helsinki, Finland

**Keywords:** agency, disability, identity, narrative positioning, non-standard employment, work ability support

## Abstract

This study aimed to explore the work ability support experiences of workers with disabilities in non-standard employment (NSE) from the perspective of agency and identity work. As extending working lives becomes crucial in Western societies, workplaces are implementing disability management programs and support measures. While organizational support like work accommodations benefits both employees and employers, workers in NSE may lack access to such support at workplaces. Using narrative positioning analysis and treating interviews as social interactions where the knowledge is co-created by the interviewer and interviewee, we show how agency and identity are negotiated in relation to employer-based work ability support and how identity positions available and deployed for the interview participants are jointly constructed during the interview process. We present our results through 3 narrative types: the self-reliant and resourceful worker, the inadequate and vulnerable worker and the disabled but good worker. Our analysis highlights workers’ own voices and situationally constructed and nuanced agency and identity. Based on our findings, we suggest that in maintaining work ability in NSE, the emphasis is on individual responsibility, resources and actions, requiring significant identity work. This identity work is shaped by contemporary worker ideals, influenced by ableist and neoliberal norms.

## Introduction

Supporting individuals’ work ability to extend their careers has become crucial in Western societies due to an aging workforce.^1-3^ Many countries have enacted societal changes, including active welfare programs and legislative reforms, to improve work participation and direct stakeholders (eg, employers, occupational healthcare and employment) toward enhancing workforce integration.^4-7^ Encouraging individuals to participate in the workforce according to their abilities and recognizing work ability as a valuable asset has therefore gained popularity.^8^

In Finland, which is the context of our study, work ability support largely relies on employer actions. Supporting employees’ work ability and preventing disability is an employer duty based on law, and systematic and goal-oriented programs for this are recommended.^7,9,10^ Programs aimed at work disability management are complex and may address various aspects of work disability prevention. However, their primary goal is to facilitate staying at work and return to work for employees experiencing temporary or permanent disabilities.^11^ In Finland, employers typically plan disability management programs in collaboration with occupational healthcare, as legislation mandates the provision of preventive occupational healthcare and cooperation to prevent work disability.^12^ These programs encompass joint actions and practices with occupational healthcare, as well as employer-initiated measures to support work ability. Even when occupational healthcare participates in supportive measures for an employee, such as by recommending workplace accommodations, the employer retains the ultimate responsibility and authority to decide whether these recommendations will be implemented. Our focus in this study was on employees’ subjective experiences with actions taken by employers to support their work ability at workplaces.^13^

Overall, employer-based supportive practices, such as work accommodations, can reduce sickness absences, minimize productivity loss without raising costs, and enhance sustainable employment for individuals with disabilities.^14-21^ However, factors like economic considerations influence the level of workplace support for an individual employee, with employers more likely to engage in support activities based on the perceived value of an employee.^22^ It has been suggested that modern working life, characterized by flexibility, have led to transactional employment relationships focused on immediate worker value and productivity.^22,23^

Our study focused on experiences on workplace support for work ability regarding workers in non-standard employment (NSE), a phenomenon partly stemming from increased labor market flexibility.^24^ NSE encompasses work arrangements that differ from the typical model of regular, permanent employment; while definitions vary, it commonly includes fixed-term contracts, part-time jobs, temporary agency work and 0-h contracts.^24,25^ Workers in NSE may also hold multiple jobs simultaneously.^26^ NSE has been associated with employment insecurity and adverse health effects, and the Western welfare regimes are not adequately designed to address its diversity.^27,28^ However, not all workers in NSE are in precarious positions; some may voluntarily choose temporary employment or part-time work in stable conditions.^24^ There is some evidence that suggests limited opportunities for work accommodations and low return-to-work expectations for workers in NSE,^29^ and less likely accommodations due to disabilities for workers in NSE in precarious positions despite legislation.^30^ To our knowledge, no prior research has explored personal experiences of workers in NSE regarding work ability support in the workplace.

Policies and practices aimed at increasing work participation often expect individuals to be “active” participants.^4^ Flexible labor markets may require employees to meet work demands despite illness or disability, rather than workplaces accommodating them.^23^ From an “active” perspective, maintaining work ability and seeking workforce participation, even when disabled or unemployed, are viewed as individual responsibilities.^23,31^ Workplace support for work ability may depend on an employee’s capacity to take responsibility and adapt to changes.^23^ Policies emphasizing activity and self-responsibility promote an identity of managing life efficiently for societal benefit, framing passivity as deviance.^32,33^ This aligns with the concept of the “ideal worker,” who is capable, available, flexible and self-managing.^34,35^

In this study, we examined employees’ experiences of work ability support at workplaces in the Finnish retail trade, and food and restaurant services, which are low-wage service industries commonly using NSE.^36^ Given the Finnish employers’ obligation to provide work ability support for employees, our focus was on the work ability support experiences of workers in NSE, particularly in light of the growing emphasis on self-responsibility for maintaining work ability and employment even in cases of having a disability. By “disability,” we mean an illness or disease affecting temporarily or permanently individual’s ability to continue working or return to work.

## Agency and Identity in Interactions

We chose to explore the experiences of workers in NSE with disabilities from the perspectives of agency and identity work. Following Eteläpelto et al,^37^ we understand agency from a subject-centered, socio-cultural perspective, seeing it as the capacity of individuals to act, make decisions and influence their professional identity and work practices. Research typically highlights transformative agency, defined as the ability to change the structures one is part of.^38^ From a subject-centered view, individuals affect their lives and future paths through transformative actions.^39^ In contrast to transformative agency which aims at changing things, small agency has been described, for example, as enduring or adapting to difficult situations.^40^ Resistance, another form of agency, involves a complex interaction with control and emerges from norms creating tensions between different interests, often manifested through complaints about work or demands for higher compensation.^40-42^

The socio-cultural perspective on agency highlights the interplay between identity and agency, emphasizing the role of social and cultural factors, available resources and personal life experiences in shaping professional development and actions.^37^ Agency is seen as a discursively and socially constructed phenomenon, which manifests in diverse positions in discourse and interaction and is shaped through negotiation within social resources. Identity positions are seen as referring to the ways individuals define and perceive themselves within various roles and contexts, influenced by social, cultural and professional environments. They are dynamic perspectives adopted in different situations, subject to change based on external environments. Thus, agency involves the ability to modify or reinforce these identity positions, that is, how and what positions individuals can adopt.

By examining identity positions in our interview data, we explored how workers with disabilities in NSE construct their identities and agency through these interactions.^43^ The subject-centered, life-course and sociocultural perspective on agency acknowledges both individual participation and sociocultural influences,^37^ suggesting that the interviewee (or other agentive subject) actively constructs identity positions using available dominant discourses, while also being somewhat constrained by them. This connects micro-level individual actions with macro-level social structures.^43^ Additionally, the interaction context influences how these identity positions are constructed. Viewing interviews as social situations where identities and agency are collaboratively constructed by both the interviewer and interviewee,^44^ we recognized the interviewer’s role in shaping and negotiating them. This will be further detailed in Methods Section.

In our study, agency and identity positions are relevant in 2 ways, as highlighted in the literature. First, given that workers with disabilities in NSE may lack the same access to work ability support (eg, due to less likely opportunities for work accommodations and low return-to-work expectations^29,30^) as those in standard employment and face greater employment insecurity, their own agency may be crucial for achieving sustainable employment. Second, the neoliberal “active” welfare state logic emphasizes individual responsibility,^4,45^ requiring individuals to maintain work ability and continue working despite disabilities. While this approach can empower individuals and offer inclusion opportunities, it can also lead to inequalities by placing more self-responsibility on individuals.^46^ Viewing work ability as a personal project necessitates new forms of agency in the working life.^31^ As individuals are expected to actively manage and shape their careers through their own agency, their identity constructions at work become increasingly important. This ongoing re-negotiation and re-shaping of identities is a key feature of modern working life.^37^

We aimed to answer the following questions: (i) How and what kind of identity positions for employees with disabilities in NSE are constructed and negotiated in interviews related to work ability support at workplaces? (ii) What kinds of working life agencies are expressed and negotiated for workers in NSE with disabilities?

## Methods

This qualitative study was conducted in Finland between September 2022 and March 2024 as a part of a larger research project focusing on multiple jobholders, who often work in NSE.^47^ Two research articles have been previously published from the project, one focusing on the career paths of multiple jobholders^48^ and one on the system perspective of work ability support and workplace safety for multiple jobholders.^49^ We used SRQR as a guideline in conducting this study.^50^ Our data in this study consisted of 11 thematic interviews with workers in the Finnish retail trade and restaurant and food service industry, collected either face-to-face or online, using Microsoft Teams. The interviews lasted approximately an hour each and were recorded with an external recorder to ensure security of the data. The recordings were transcribed verbatim by a purchased service, totaling 201 pages of transcriptions. To ensure accuracy, the transcriptions were checked against the recordings. Approval of the ethical committee of Finnish Institute of Occupational Health was obtained. All participants provided informed written or recorded consent. When verbal consent was recorded instead of written consent, interviewees confirmed that they had read the information about the research and their rights, were given the opportunity to ask questions and then provided their consent to participate in the study.

We recruited participants from a survey in the larger research project, selecting those who expressed willingness to be interviewed and provided their email addresses. Out of 32 employees contacted, 11 participated in interviews. By the 11th interview, no new insights relevant to the research questions were formulated, suggesting that data saturation had been achieved. The study included working-aged women and men from various occupations, including retail workers, kitchen staff, bartenders, waitresses and chefs, who had experience of working in industries using NSE often held multiple jobs. Further demographic information was not collected and thus cannot be reported. This decision was grounded in both analytical and ethical considerations. Our theoretical and methodological approach, narrative positioning analysis, examines how speakers position themselves and others linguistically and contextually, viewing identity as constructed and dynamic. This inductive, data-driven analysis emphasizes the internal meanings of narratives, making external demographic variables non-essential. Additionally, not collecting demographic details ensured better protection of participant anonymity.

Two of the 11 interviewees were excluded from this study because they did not mention impairments or disabilities affecting their work, focusing instead on the 9 who did. As we focused on interviewees’ perspectives and experiences regarding work ability and support, rather than asking for detailed health information, we included any discussion of challenges or difficulties completing work tasks due to self-assessed health conditions in our analysis.

The interview themes are presented in a Supplemental File. Interviews were conducted flexibly elaborating topics when needed to gain deeper insights from interviewees’ answers. Themes included work ability related topics such as work ability in general, working arrangements, support and cooperation, having multiple jobs simultaneously and career development and prospects.

We applied narrative positioning analysis to investigate the NSE workers’ experiences of work ability support at workplaces, since it allows for analyzing the construction of identity and agency in relation to cultural model stories.^43^ Narrative positioning analysis is conducted on 3 intertwined levels, presented in Table 1, aiming to connect the individual and sociocultural levels.^43^ Although the analytical steps are described as distinct, mobility between stages is necessary to fully understand the teller’s positions and their links to cultural model stories.^51^

**Table 1.** Levels of Positioning Analysis Adapted from Bamberg.^43^

Level	Characters	Explanation and examples
Level 1: Story world	How are the characters positioned within the story	Describing self in relation to others within the story, eg, receiving help from co-workers, suffering from negative employer attitudes
Level 2: Storytelling	How is the narrator positioning himself/herself (and is positioned) within the interaction situation	Narrating as an activity, eg, the interview questions and responses, follow-up questions, acknowledgment tokens
Level 3: Cultural model stories	How the narrator positions a sense of self in relation to dominant discourses	Presenting oneself as a particular kind of person, eg, a good employee, a pretender

In order to examine the ways in which the identities and agencies were constructed as mutual accomplishments by the interviewer and interviewee we applied tools of conversation analysis to the storytelling level.^52,53^ Narrative positioning analysis orients to the analysis of the storytelling level as a situation of social interaction similarly to conversation analytic approach, examining the ways in which the stories are told. Interviews can be seen as situations in which the knowledge created is a mutual interactional achievement of the interviewer and the interviewee, meaning that by interview questions and responses to the storytelling, the interviewer becomes a co-author and participates in negotiations of identity positions.^44^ Conversation analysis for its part focuses on analyzing the structures of interaction, offering tools for examining the turns of speech and other features of conversation.^52,53^

Our team consisted of researchers with expertise on occupational healthcare, sociology, education and social psychology. Our analytical process began by listening to the recordings and thoroughly reading once all the interview transcripts. Through an iterative process, we identified segments where interviewees shared stories about how their impairments or disabilities affected their work ability and their experiences with support at their workplaces. We then analyzed each interview segment individually, focusing on identifying the story characters and their roles in relation to work ability support in the workplace. At the story world level, we identified characters such as employees, employers and co-workers, and explored their relationships. We then advanced to the storytelling level, analyzing how identity positions were jointly co-constructed by the interviewer and the interviewee. We examined how interviewees shared their stories with interviewers, paying attention to the interaction between them, including the design of and response to questions, as well as the interviewer’s reactions and conversational elements like laughter and intonation. In the cultural model story level, we reviewed story segments and worked comparatively to identify recurring dominant discourses. This phase was closely linked to earlier analyses, focusing on how these model stories were invoked and made relevant in stories and storytelling. We examined how these model stories shaped identity positions and expressions of agency, and how they were negotiated as shared cultural knowledge.

## Results

**Figure 1.**
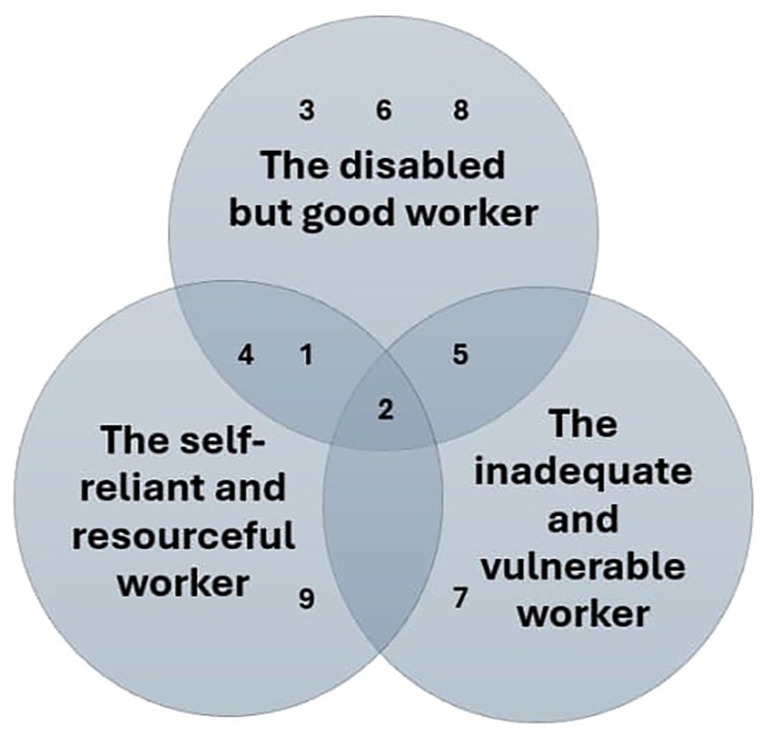
Narrative types and their distributions.

In this section, we present our findings through 3 narrative types we found. Figure 1 shows the narrative types and their distribution in our data. However, this representation is simplified, as narratives are constructed in a multidimensional manner, and the same forms of positioning may appear across different accounts in varying ways, contexts and narrative tones. During the analysis, it became evident that these forms of positioning do not distribute neatly across individual cases but instead form overlapping and intersecting structures of meaning.

Since narrative positioning analysis emphasizes the qualitative depth and richness of individual stories rather than the quantitative distribution of narrative types, we will present our findings through an in-depth analysis of 3 selected excerpts from our data. The first narrative type (*the disabled but good worker*) illustrates an employee who expresses transformative agency while adopting the negative identity position of a disabled worker. Although expressions of transformative agency were common in our data, they often involved individuals taking personal responsibility, such as changing jobs to better suit their abilities. This narrative demonstrates how employees, even when receiving work ability support at workplace, constructed disability as an undesirable characteristic. Throughout the dataset, disability was consistently constructed as undesirable, contrasting with the cultural model story of an ideal worker. Interviewees typically portrayed themselves as good workers by downplaying the impact of their disability and emphasizing their positive qualities as employees.

The second and third narrative types (*the self-reliant and resourceful worker* and *the inadequate and vulnerable worker*) show 2 extreme cases. The former shows a case where an employee with disabilities rejects the identity position of a disabled worker and challenges the traditional power dynamic between employee and employer, thus expressing powerful transformative agency and resistance. The latter illustrates an extreme case from the opposite end, with precarious positioning and expressions of small agency. The extreme cases serve in strengthening our analytical points,^54^ illustrating in-depth insights of how cultural model stories invoked shape the identities and agencies of the interviewed employees. In terms of various forms of agency, they naturally overlapped and were expressed simultaneously or periodically^40^ in the employees’ stories, but the chosen excerpts highlight the certain forms of agency and their relationship to dominant discourses.

### The Disabled But Good Worker

**Figure 2.**
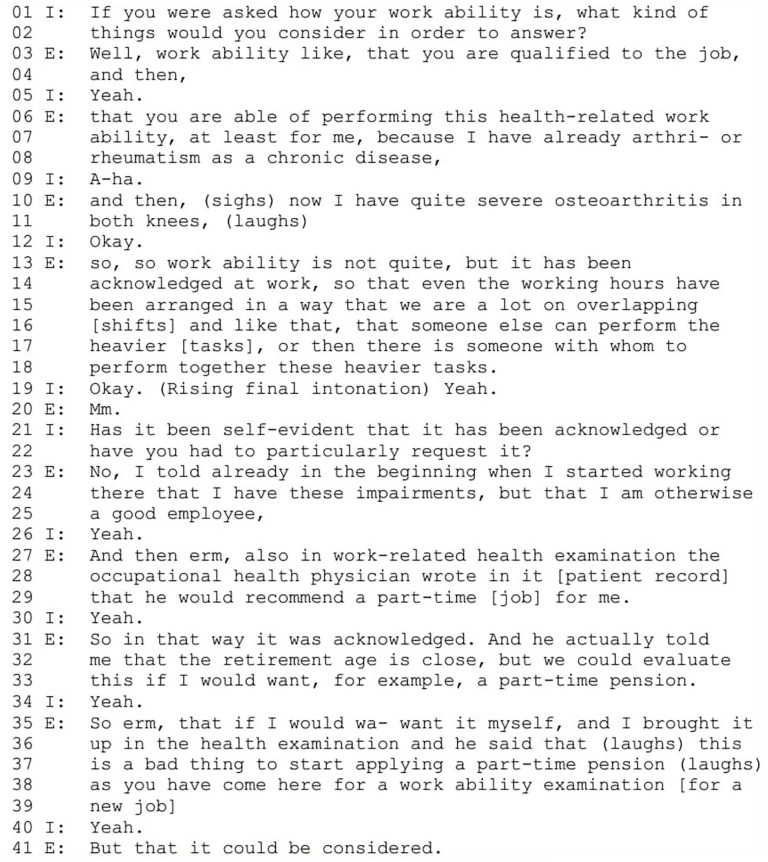
Excerpt 1.

In this narrative type, employees position themselves as temporarily or permanently disabled and describe occasional experiences of work ability support at workplace. They express transformative agency by disclosing their disability to their employers and requesting support. The disabled worker identity is constructed as undesirable, while other characteristics of a good worker, such as positive attitudes and lack of sickness absences, are emphasized. Excerpt 1 (Figure 2) is from an interview with a near-retirement-aged employee working part-time as a salesperson in retail and additional hours via a temporary work agency in the same industry (E = employee, I = Interviewer).

At the story world level, the characters include the employee, employer representatives, co-workers and an occupational health physician. The employee first discusses work ability in general (from line 3 onward), and then positions herself as disabled by mentioning her severe musculoskeletal diseases (“rheumatism,” line 8 and “severe osteoarthritis” line 10) that affect her work ability. She describes how her situation has been acknowledged at work, with employer representatives referred implicitly in passive and co-workers taking on tasks she cannot perform (lines 13-18). By referring to indefinite employer representatives, she constructs accommodations as a neutral part of workplace practices, although this neutrality is later contradicted in her story. The employee contrasts the identity position of a disabled worker with that of a good worker, constructing disability as undesirable (“but I am otherwise a good employee,” lines 24-25). She then introduces the occupational health physician as a new character in her story, describing the physician recommending part-time work for her (lines 27-29). She implies her own agency by framing the possibility of applying for a part-time disability pension as depending on her own willingness (lines 33 and 35). The disability is repeatedly constructed as an undesirable characteristic on a worker, as the employee describes the occupational physician’s view that applying for the pension is a “bad thing” when starting a new job (line 37).

At the storytelling level, the employee’s story is a response to the interviewer’s question (lines 1-2). As the storytelling goes on, the interviewer produces acknowledgment tokens (“yeah,” “a-ha”) to encourage the employee to continue. When the employee describes accommodations made in her work, the interviewer’s interest is indicated by “okay” (line 19) with rising final intonation, suggesting the topic’s importance.^55^ The interviewer further confirms the topic’s relevance by asking if work arrangements were self-evident or specifically requested (lines 21 and 22), thus becoming a co-author in the storytelling.^44^ The employee’s response is designed in the way that shows her orientation rather to her entitlements of work accommodations than on who initiated the process. First, by describing how she brought up her disability early in employment and constructing an identity position of a disabled but good worker (lines 24 and 25), she implies openness about her disability and justifies the need for accommodations by appealing to her positive characteristics. Second, she references a medical expert’s evaluation of her declined work ability (lines 25-27) to legitimize the need for work accommodations, suggesting the issue is not unproblematic and may require justification.^56^

At the cultural model story level, the contradictory identity position the employee constructs, includes a negative identity as a disabled worker with lower value and a positive identity as an “otherwise” good worker, where goodness exists despite the disability. This reinforces the cultural model of an ideal worker by downplaying disability and emphasizing other positive characteristics. By articulating the justifications for her work accommodations, she distinguishes herself from someone who might seek adjustments without valid reasoning. She thereby distances herself from the negation of ideal worker, who is unwilling to perform certain work tasks.

### The Self-Reliant and Resourceful Worker

**Figure 3.**
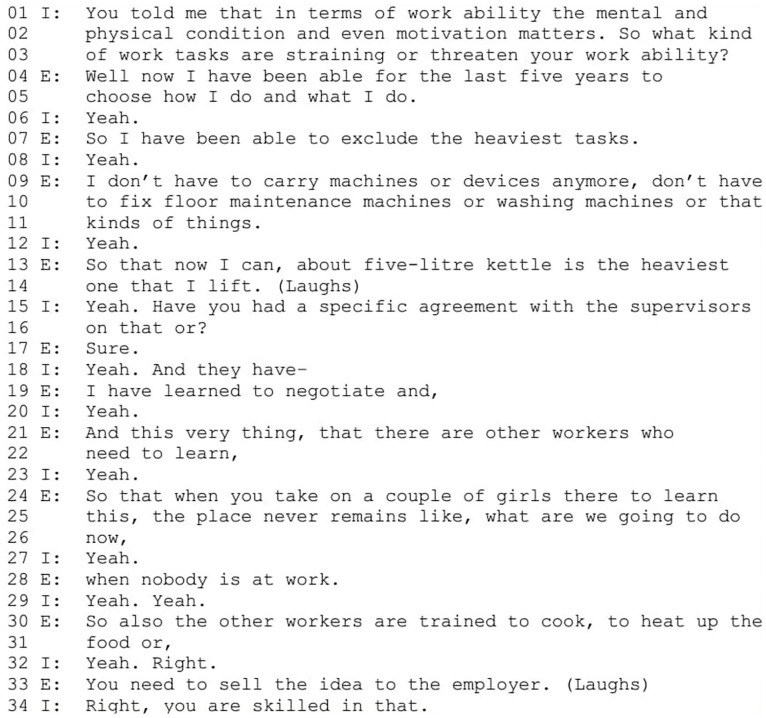
Excerpt 2.

In this narrative type, employees position themselves as strong agents in managing their work ability and necessary adjustments. Excerpt 2 (Figure 3) demonstrates an extreme case in our data where the employee, while constructing herself as self-reliant and resourceful worker with strong transformative agency, challenges also the traditional view of employer-employee power relationship. This employee, with extensive job experience including cleaning and food services, now works as a care assistant in a nursing home, also preparing the residents’ food. She has no vocational training or degrees. She has had surgery on both hands, limiting her ability to perform in her previous, physically demanding jobs, such as cleaning and large-scale cooking (E = Employee, I = Interviewer).

At the story world level, the employee frames the story around her capabilities, self-reliance, and resources in making changes, expressing transformative agency. Characters in this story include the employee, the supervisors and young co-workers referred to as “girls.” The story begins with the employee describing her possibility to choose suitable work tasks for her work ability (from line 4 onward), positioning herself as having special rights to opt out of certain tasks. The interviewer acknowledges this and introduces supervisors as new characters in the story asking whether the work accommodations were agreed upon (lines 15 and 16). The employee affirms this (“sure,” line 17), acknowledging the supervisors’ influence in the matter but continues then with a story about her own negotiating skills (line 20). Her transformative agency is further expressed later when she positions herself as advising the employer on arranging the work (line 33).

At the storytelling level, the employee takes the position of an interviewee by answering the interviewer’s questions but steers the conversation with her responses toward her possibilities and agency. She resists the identity position of a disabled worker suggested by the interviewer in her question (lines 1-3), by developing the topic from straining tasks to her own possibility to choose in her response (from line 4 onward). When the interviewer asks about work arrangements, presupposing an agreement with the employer is needed (lines 15 and 16), the employee contradicts the subordinate position implied by the interviewer. She confirms the presupposition with “sure” (line 17), which marks the positive response as self-evident.^57^ She then interrupts the interviewer’s follow-up question (“and they have-,” line 18), and frames the agreement as a result of her own negotiation skills (line 19). The employee then shifts away from the story world elaborating on the terms of the negotiation, implying her own impact on arranging the work (from line 21 onward). With laughter, she decreases the seriousness of her claim, as implying that an employee influencing recruitment policy of a workplace might be viewed as problematic.^44,58^ The laughter invites the interviewer to respond, and she affiliates with the employee’s turn, confirming the employee’s construction of herself (“Right, you are skilled in that,” line 34). Thus, the employee’s position as self-reliant and resourceful worker with strong agency is co-constructed by both the employee and the interviewer.

At the cultural model story level, she resists the position of a worker with lower capacity, implying that her disabilities are irrelevant. Instead, she emphasizes her other resources, such as negotiation skills and ideas for organizing work. She expresses powerful transformative agency in making changes and influencing work arrangements, and resistance in constructing power relations between employee and employer in a way that deviates from the traditional cultural model story. By doing so, she resists the idea of the (disabled) employee as the more vulnerable one. However, her distancing from the position of a disabled and vulnerable worker, coupled with her emphasis on self-reliance and resourcefulness, suggests that she takes responsibility for her disability-related limitations as an employee and compensates for them with her skills. Ultimately, she reinforces the neoliberal cultural model story of a self-responsible and creative worker.

### The Inadequate and Vulnerable Worker

**Figure 4.**
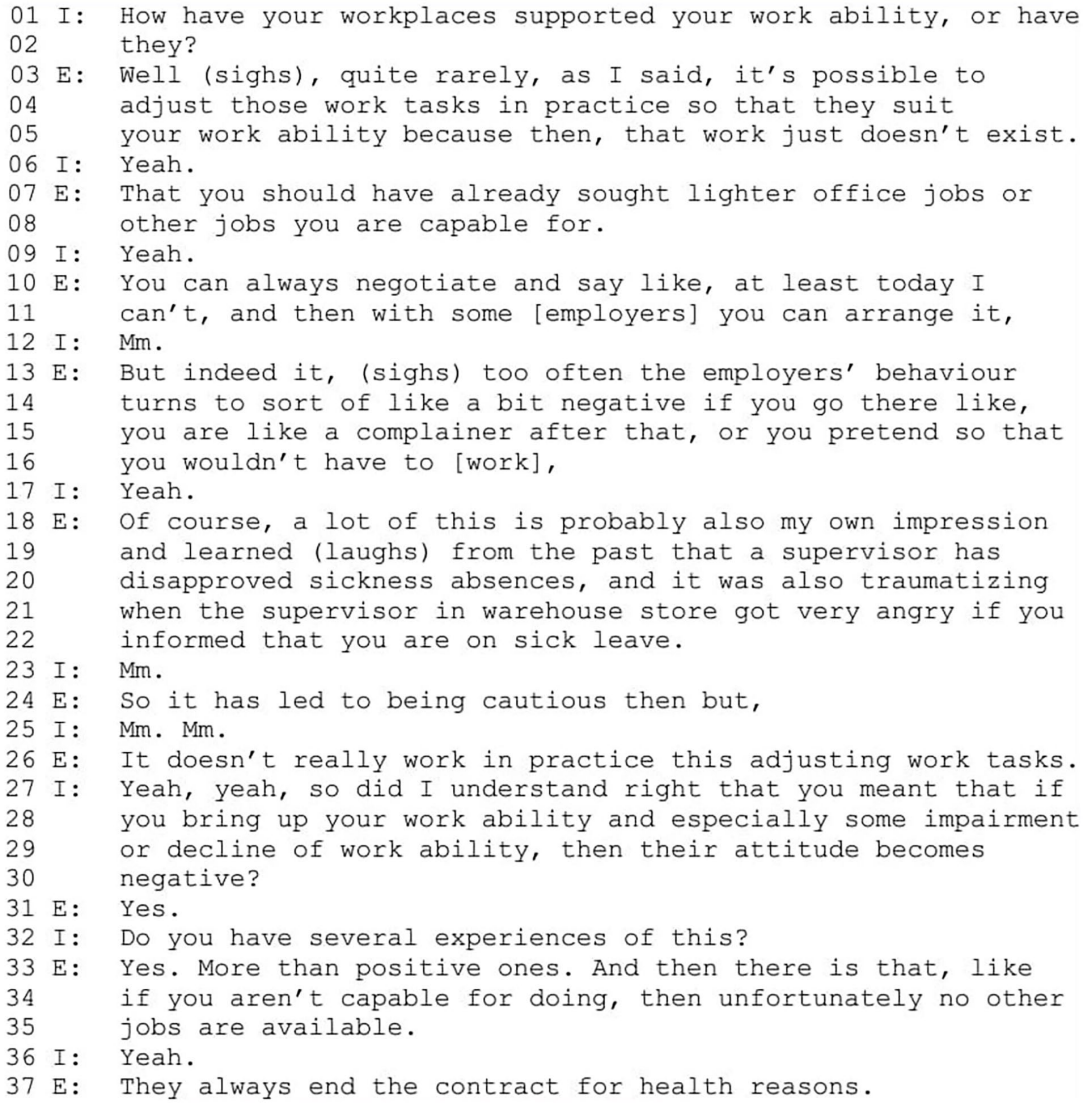
Excerpt 3.

In this narrative type, employees construct themselves as weak agents with limited possibilities. They position themselves as victims of excessive job demands, such as being forced to work when sick, unable to adjust their work according to their health or being rejected due to disabilities. Excerpt 3 (Figure 4) illustrates an extreme case of this narrative. This excerpt is from an interview with an employee in her forties who works in seasonal retail with a 2-month contract at the time of the interview. Throughout her career, she has worked mostly in various low-wage industries, including retail, and has no vocational training or degrees. Her family background includes a history of addiction and poverty, leading her to start working very young instead of obtaining an education. She has a chronic musculoskeletal illness, among other conditions affecting her work ability (E = Employee, I = Interviewer).

At the story world level, the characters are the employee herself, employers in general and a specific supervisor from a past workplace. The employee’s story is framed around a lack of possibilities in life. She begins by stating that work accommodations are rarely possible because such jobs do not exist in organizations (lines 3-5). She then refers to her lost possibilities from a life-course perspective (“you should have already sought,” line 7), positioning herself as an employee without education and without opportunities to obtain one, thereby implying limited opportunities for physically lighter jobs for her. With this, she positions herself as responsible for finding a suitable job for her work ability but also as a socioeconomically vulnerable victim of circumstances. By referring to non-existence of suitable jobs, she positions employers as incapable of affecting the situation.

Next, the employee’s positioning of employers in her story changes from incapable to unwilling. She refers to the possibility to negotiate work accommodations in some extent (lines 10 and 11) but constructs most employers as unwilling to acknowledge her declined work ability (lines 13-15). The employers are positioned as antagonists, denying her opportunities to work and treating employees as “complainers” or “pretending” (lines 15 and 16). By using the categories of a complainer and a pretender, the employee constructs employers in her story as viewing disabled employees, herself included, as having unwarranted complaints or requests.^44^ The employee describes how previous employers’ negative attitudes toward sickness absences have influenced her own actions (“so it has led to being cautious then but,” line 24). By this, she expresses small agency in enduring negative aspects of working life and adjusting her attitude accordingly. She concludes by stating that accommodating work generally does not work (line 26). After the interviewer’s clarifying question, she reiterates that alternative jobs are simply not available (lines 33-35) and contracts end because of health issues (line 37). In sum, the employee positions herself as an inadequate and vulnerable worker establishing a power relationship between her and employers, and between her and labor market structures with her in subordinate positions.

At the storytelling level, the employee and the interviewer both participate in constructing the position of vulnerable worker. The employee begins her story as a response to the interviewer’s question, and the interviewer responds with minimal responses (“mm,” “yeah”) showing acknowledgment and encouraging the employee to continue. The employee then momentarily turns away from the story world by commenting the subjectivity of her story (lines 18-19). She had previously generalized the employers’ behavior (“too often,” line 13), but now frames her story as her own “impressions” and “learned” experiences (lines 18 and 19). By presenting her view as based on individual experiences and adding laughter (line 19) she manages the subjectivity of her story and distances herself from overgeneralization.^59^ She continues her story by describing a past employer’s poor behavior (lines 20-22) to further illustrate her negative experiences. The interviewer poses a clarifying question, formulating the gist^60^ of the story as being about employers’ negative attitudes toward employees raising work ability issues (lines 27-30). After the employee confirms this (“yes,” line 31), the interviewer continues with a follow-up question concerning the repetitiveness of her experience (line 32), thereby acknowledging the suffering and vulnerability as a worker in the employee’s story and seeks confirmation. The employee confirms and further develops this identity position of a vulnerable by mentioning employers ending contracts due to health issues (line 37), legitimizing her claim with the extreme case formulation “always.”^61^

At the cultural model story level, 2 dominant discourses are invoked. The first is about a worker who is vulnerable due to disability, socioeconomical background and the attitudes of working life toward disabled workers. This identity position is co-constructed by the interviewer and the employee, and it reflects the socio-economical and structural aspects of inaccessibility to work ability support measures in the workplace. The second is the story of an inadequate worker in terms of work performance and education, bringing only limited value to employers. This is the negation of an ideal worker who is capable, motivated, and valuable to employer. The employee adopts this negatively valued identity position consisting of neoliberal and ableist norms and constructs the employers’ negative attitudes and behaviors deriving from her inadequacy as a worker. Her small agency emerges from her embracing this identity, enduring the employers’ attitudes and adjusting her own behavior according to these norms.

## Discussion

This study aimed to shed light on the NSE workers experiences of work ability support at workplaces. By using narrative positioning analysis and treating interviews as social interactions where the knowledge created is a mutual accomplishment of interviewer and interviewee, we demonstrated how these workers negotiate their agency and identity in relation to this support. We also illustrated how identity positions are jointly constructed during the interview process. Our findings suggest that regarding the experiences of maintaining work ability in NSE, the emphasis is on individual responsibility, resources and actions, requiring significant identity work. The ableist and neoliberal ideal worker identity was perceived as the norm,^34,35^ shaping how stories of work ability support were constructed.

Considering the limited experiences of actual support at workplaces and the stories of job transitions in our data, our findings align with previous studies^29,30^ showing that workers in NSE may lack access to supportive measures at their workplaces in case of disability. Our study also underscores the importance of employees’ own actions and resources in acquiring support or more suitable jobs. Adopting a subject-centered sociocultural perspective on agency as suggested by Eteläpelto et al^37^ and using the multileveled analytic orientation of narrative positioning enabled us to demonstrate how self-responsibility can serve both as means for workers in NSE to personally manage their working conditions and as a constraint imposed by sociocultural factors.

Most workers presented themselves as capable for influencing their work situations in case of illness or disability. They reported ways such as disclosing disabilities to employers, requesting accommodations or changing jobs to better suit their abilities, thus expressing transformative agency. Actual support from employers, like work accommodations, was infrequently experienced. Our study suggests, that in-firm transformative agency, as defined by Järvensivu,^40^ may not be that common for NSE workers.

Regarding in-career transformative agency,^40^ job changes were common, suggesting that flexible labor markets provide opportunities for workers to find roles that meet their needs. This aligns with the notion that NSE workers are a heterogeneous group and not all in precarious situations: All forms of non-standard employment are not precarious, and there are other aspects than the form of employment contributing to precariousness. Precarious employment essentially involves transferring risk from employers to workers. This includes income and employment insecurity, along with a lack of workplace rights and social protections, which shift financial and occupational health and safety risks onto the employees.^25^ Previous studies indicate that while some workers with disabilities may prefer NSE for its flexibility, health-related reasons often lead to more precarious work types.^62^ Thus, while job mobility is important for employees with disabilities in contemporary labor markets and especially in NSE, stories of job changes may also indicate that the responsibility for finding suitable work largely falls on the employees.

By applying narrative positioning analysis, we were able to reveal cultural model stories – or dominant discourses – related to work ability support at workplaces for workers in NSE. As Eteläpelto et al^37^ note, the agency and identity are always negotiated within the limits of wider socioeconomical and material constraints and resources. As we showed, the interviewed employees used the cultural model story of an ideal worker as a resource in their stories about them navigating in NSE with disabilities. All interviewed employees viewed disability as a characteristic that violated the ideal of a good worker, and it was common to construct oneself as a good worker by downplaying the impact of their disabilities on their work and emphasizing their positive worker characteristics to compensate for their limitations. This aligns with previous studies^63,64^ which note that disability identity carries stigma in in the context of contemporary working life, making creating a positive work-related identity both a strategy and a challenge for workers with disability.

The contemporary society’s cultural model story of a self-responsible, resourceful and adapting employee^35^ served as a resource and a constraint to the interviewed employees in their negotiations of agency and identity. Our extreme cases highlight these 2 contrasting ways the model story of an ideal worker can function. First, as our second narrative type (the self-reliant and resourceful worker) showed, by rejecting the identity position of a disabled and potentially vulnerable worker, and instead adopting an identity as self-reliant, creative, resourceful, and adaptive, workers with disabilities can present themselves as powerful transformative agents within the organization. As demonstrated in our second narrative type, the employees may also create resistance by enacting an alternative cultural norm, one where workers craft their own work conditions rather than relying on traditional employer-employee power dynamics.^42^ This self-responsible and resourceful identity aligns with the contemporary worker ideals^35^ and embracing this neoliberal norm can be seen as empowering and allowing the individuals to influence their work conditions differently than through traditional power structures. However, in terms of work ability support at workplaces, such employees may end up adopting responsibility for their disability and its effects on work, as well as for finding solutions independently.

Second, our third narrative type (the inadequate and vulnerable worker) illustrated the opposite end, that is, limited agency. In a socioeconomically vulnerable labor market position where the worker with disabilities is forced to choose NSE and is left without support, agency becomes restricted, especially for those lacking cultural, economic and social resources.^65,66^ In the third narrative type, employee constructed an identity position marginalizing them as unable to meet the ableist ideal,^67^ crafting thus a negation of the cultural model story of an ideal worker for herself. At the same time, that exact ideal became reinforced. Järvensivu^40^ has found that a restricted form of agency may manifest as small agency. As demonstrated in the third narrative type, the employees expressed small agency by adopting an identity position of an inadequate worker, accepting her own treatment as inadequate in terms of labor market, enduring suffering and silently adapting one’s behavior and attitude. As Jammaers and Zanoni^67^ note, adopting a negatively valued identity may suggest that workers with disabilities are willing to accept identity positions that are not valued to continue participating in working life.

Our findings suggest that workers in NSE with disabilities may conform to the neoliberal and ableist norms of the contemporary worker ideal. By crafting positive identities that emphasize their value despite disabilities, they inadvertently reinforce ableism as a foundational principle in the workplace,^63^ thereby supporting the very structures that oppress them. This also reinforces the discourse of employees with disabilities not being ideal workers, even though for these employees, developing a positive identity can be seen as a tool for rejecting the lower-value identity of a disabled worker.^63^ Rejecting lower-value identities may be crucial for employees, as research shows that employer engagement in work ability support varies based on perceived employee value.^22^ Previous studies^63,67^ on employees with disabilities indicate that they might resist ableist narratives and highlight positive traits derived from their disabilities, such as empathy, to create a positive identity. However, in our study concerning employees with disabilities and working in NSE did not find such positionings. Instead, the identity of a disabled worker was consistently constructed negatively.

In this study, we have also demonstrated how a detailed analysis of discursive features further elaborates the sharedness of the identity positions available and deployed. Narrative positioning analysis enables the exploration of the identity positionings and agencies constructed in interviewees’ stories, and the second level of analysis also acknowledges how the interview context shapes and is shaped by the narratives available and deployed.^68,69^ This means approaching the interview as an occurrence that is defined by the interaction participants in situ rather than only as a way for a researcher to gather information and understanding that knowledge created is a mutual interactional achievement of the interviewer and the interviewee.^44,68^ To further deepen this level of analysis, we engaged in a thorough and systematic analysis of the interview situations by applying aspects of conversation analysis, such as examining turn-taking, turn construction and small details of conversation, such as laughter or intonation. Instead of treating them as irrelevant parts of data, we included also the interviewers’ questions and responses, even minimal ones like “a-ha” or “yeah,” in the analysis. This way we were able to show explicitly how identity positions are accomplished jointly during the interview. As we showed, the interviewers’ questions may make certain topics more relevant than others and propose certain identity positions, and the interviewees for their part may accept or reject these identity positions or invite interviewers to affirm an identity position they constructed in their stories. We suggest that this approach reinforces the analytic interpretations made and demonstrates how the available cultural model stories are recognized and shared, even by the interviewers themselves.

Our study is limited by its small sample size. While small sample size is recommended for narrative research to enable rich, in-depth analysis,^70^ it may affect the transferability of our findings. We aimed at rich and thick descriptions in reporting our findings so that the readers can determine the transferability of the findings.^70^ Conducted within the Finnish retail, food, and restaurant sectors and cultural context, our findings may not be applicable to other countries or industries. Future research could use larger sample sizes and cross-cultural comparisons to further validate and expand our conclusions.

## Conclusion

Our findings align with previous research suggesting that workers in NSE may have limited access to workplace support. However, a more significant finding of this study is that NSE workers with disabilities prioritize individual actions, resources and career-based agency over organizational support, even in a system like Finland’s, which is in many ways reliant on employer actions in the workplace. Future investigations should examine whether work ability support systems for NSE workers adequately address their needs. Additionally, those involved in managing work ability issues should be trained to recognize the importance of individual responsibility, resources, and actions – which require substantial identity work – and to effectively address these aspects.

## Supplemental Material

sj-docx-1-inq-10.1177_00469580251376226 – Supplemental material for Striving for the Ideal: Narrative Positioning Analysis of Work Ability Support Experiences of Employees with Disabilities in Non-standard Employment Supplemental material, sj-docx-1-inq-10.1177_00469580251376226 for Striving for the Ideal: Narrative Positioning Analysis of Work Ability Support Experiences of Employees with Disabilities in Non-standard Employment by Hanna Keränen, Anu Järvensivu and Ritva Horppu in INQUIRY: The Journal of Health Care Organization, Provision, and Financing

sj-docx-2-inq-10.1177_00469580251376226 – Supplemental material for Striving for the Ideal: Narrative Positioning Analysis of Work Ability Support Experiences of Employees with Disabilities in Non-standard Employment Supplemental material, sj-docx-2-inq-10.1177_00469580251376226 for Striving for the Ideal: Narrative Positioning Analysis of Work Ability Support Experiences of Employees with Disabilities in Non-standard Employment by Hanna Keränen, Anu Järvensivu and Ritva Horppu in INQUIRY: The Journal of Health Care Organization, Provision, and Financing
