# Supplementary material for: Striving for the Ideal: Narrative Positioning Analysis of Work Ability Support Experiences of Employees with Disabilities in Non-standard Employment
Source: Inquiry. 2025 Sep 22;62:00469580251376226. doi: 10.1177/00469580251376226 (PMC12454955; doi:10.1177/00469580251376226)
Supplement: sj-docx-2-inq-10.1177_00469580251376226 – Supplemental material for Striving for the Ideal: Narrative Positioning Analysis of Work Ability Support Experiences of Employees with Disabilities in Non-standard Employment [file sj-docx-2-inq-10.1177_00469580251376226.docx]

**Additional file 2. Interview guide**

**Interview guide for multiple job holders**

Background

- What kind of jobs do you do?
- How many employers do you work for (or are you self-employed)? How are your working hours distributed among different workplaces?
- What are your reasons for working multiple jobs?
- How long have you been working multiple jobs simultaneously?
- What are the advantages of working multiple jobs? And the disadvantages?
- Do you plan/want to continue working multiple jobs? Why or why not?

The concept of work ability

- If somebody asked you about your current work ability, what kind of issues would you consider answering the question?
- How do you determine that your work ability is good or if it has declined?
- How often do you think about your work ability? In what situations?

Maintaining and supporting work ability

- What factors affect work ability in general?
- What aspects of your work strengthen your work ability? What aspects of your work impair your work ability?
- How do your workplaces support your work ability? (More detailed questions, e.g., about orientation to work tasks and occupational health and safety; social relations in the workplace; early and enhanced work ability support; access to information and training)
- How does occupational healthcare support your work ability?
- Who is responsible for addressing issues affecting work ability?
- How do you recover during your free time? What hinders and promotes recovery?
- How well do you balance work and other aspects of life?

Is there anything else you would like to say about these themes we have discussed?
